# Beyond language: A structured profile of number processing impairment in logopenic primary progressive aphasia

**DOI:** 10.1177/13872877261450931

**Published:** 2026-05-25

**Authors:** Joël Macoir, Monica Lavoie, Robert Laforce

**Affiliations:** 1 École des Sciences de la Réadaptation, Faculté de Médecine, Université Laval, Québec, Québec, Canada; 2 Centre de Recherche CERVO—Brain Research Centre, Québec, Québec, Canada; 3 Chaire de recherche sur les aphasies primaires progressives, Fondation de la Famille Lemaire, Québec, Québec, Canada; 4 Département des Sciences Neurologiques, Clinique Interdisciplinaire de Mémoire (CIME) du CHU de Québec, Québec, Québec, Canada

**Keywords:** acalculia, Alzheimer's disease, neuropsychological tests, number processing, parietal lobe, primary progressive aphasia

## Abstract

**Background:**

The logopenic variant of primary progressive aphasia (lvPPA) is a language-led neurodegenerative syndrome commonly associated with Alzheimer's disease pathology and temporo-parietal degeneration. Although acalculia has been reported in lvPPA, numerical cognition has not been systematically investigated, and the specific profile of impairment remains poorly defined.

**Objective:**

To characterize numerical cognition impairment in lvPPA using a comprehensive, theory-driven assessment battery and to examine its clinical relevance for diagnosis and cognitive characterization.

**Methods:**

Fourteen individuals with lvPPA and twenty-eight demographically matched healthy controls completed the dCALQ, a standardized battery assessing number recognition and comprehension, number production (transcoding), and calculation. Participants also underwent global cognitive screening (Montreal Cognitive Assessment), language assessment (Detection Test for Language Impairments in Adults and the Aged), and measures of working memory and executive functioning. Group comparisons, intra-group domain analyses, correlation analyses, and receiver operating characteristic (ROC) analyses were performed.

**Results:**

Individuals with lvPPA showed significant impairments across all numerical domains compared with controls, with the most severe deficits in calculation, followed by transcoding, and milder impairment in number recognition and comprehension. Within the lvPPA group, performance differed significantly across domains, revealing a graded pattern of impairment. ROC analyses demonstrated excellent diagnostic accuracy for the dCALQ total score and strong discrimination for the calculation and transcoding domains.

**Conclusions:**

Numerical impairment is a robust and systematic feature of lvPPA rather than an incidental finding. The distinct numerical profile identified highlights the contribution of parietal-based cognitive dysfunction and supports the clinical utility of structured numerical assessment for cognitive characterization and diagnosis in dementia syndromes.

## Graphical abstract

**Graphical abstract:**
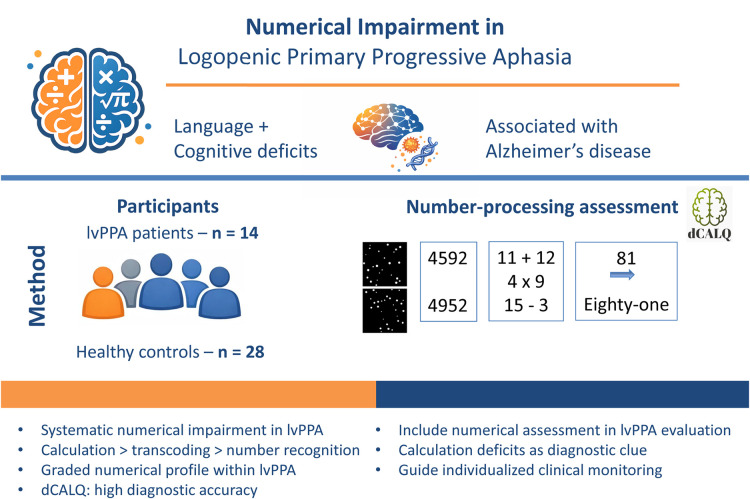
This is a visual representation of the abstract.

## Introduction

The logopenic variant of primary progressive aphasia (lvPPA) is a progressive neurodegenerative syndrome characterized primarily by language impairment, most notably anomia, phonological errors, and reduced ability to repeat sentences and verbal sequences.^1,2^ Neuroimaging studies have consistently associated lvPPA with atrophy in the left temporo-parietal junction, encompassing the angular gyrus, posterior middle temporal gyrus, and supramarginal gyrus.^3,4^ Recent multimodal imaging findings further underscore the central involvement of the left inferior parietal cortex in lvPPA. Kang et al. demonstrated that both amyloid-positive and amyloid-negative forms of lvPPA exhibit hypoperfusion and atrophy within the supramarginal and angular gyri, thereby reinforcing the view of lvPPA as a syndrome involving temporo-parietal networks that support both language functions and other cognitive processes, including numerical cognition.^5^ The underlying pathology is most frequently Alzheimer's disease with an atypical, language-dominant presentation.^6^ Although language impairment constitutes the clinical hallmark of lvPPA, additional non-linguistic cognitive deficits are increasingly recognized.^7,8^

Among these, numerical cognition is of particular interest because it depends heavily on left parietal regions, overlapping with areas typically affected in lvPPA.^9,10^ Numerical cognition refers to the set of cognitive processes that enable individuals to understand, represent, manipulate, and use numerical information.^10,11^ It encompasses abilities such as number magnitude comprehension, recognition and production of numerical symbols, arithmetic fact retrieval, calculation, and problem solving.^12,13^ Deficits in number processing and calculation, collectively referred to as acalculia, were already noted in early descriptions of the logopenic/phonological variant,^2^ and subsequent clinical observations have confirmed that numerical difficulties may accompany the broader cognitive phenotype of lvPPA.^14,15^ Importantly, however, these studies generally documented the presence of acalculia without providing a detailed characterization of the underlying numerical impairments, leaving the cognitive mechanisms largely undefined. In their comprehensive meta-analysis, Conca et al. similarly noted reduced calculation abilities in lvPPA compared to healthy controls, other PPA variants, and Alzheimer's disease, though better preserved than in posterior cortical atrophy, again without specifying the qualitative nature of these deficits.^7^ This pattern is consistent with the typical temporo-parietal involvement in lvPPA, yet distinct from parietal-variant Alzheimer's disease, in which acalculia is early, prominent, and often severe.^16^

Numerical cognition relies on a distributed fronto-parietal network supporting several components, including number magnitude comparison, symbolic transcoding, retrieval of arithmetic facts, and procedural calculation. Recent large-scale reviews and meta-analytic work have confirmed that these functions depend on coordinated activity across the intraparietal sulcus and angular gyrus, as well as prefrontal regions involved in cognitive control and working memory.^17,18^ Notably, these same parietal and fronto-parietal regions, particularly the angular and supramarginal gyri, are among the most consistently compromised in lvPPA, suggesting that degeneration of this network may directly contribute to the numerical difficulties observed in the syndrome.^3,8^ Yet no systematic investigation has characterized the specific pattern of numerical deficits in lvPPA compared to healthy individuals.

The present study aims to systematically characterize numerical cognition deficits in lvPPA using the dCALQ, a standardized and theoretically grounded screening battery that evaluates number processing and calculation across recognition and comprehension, number production, and calculation processes.^19^ By identifying the most vulnerable numerical domains and examining their relationships with language and phonological working-memory measures, this study seeks to clarify how numerical impairment fits within the broader cognitive profile of lvPPA.

## Methods

### Participants

Fourteen individuals meeting diagnostic criteria for lvPPA took part in the study (six men, eight women). Their mean age was 69.93 years (SD = 7.59), and they had, on average, 14.93 years of education (SD = 2.43). All participants were recruited through the Clinique Interdisciplinaire de Mémoire (CIME) at the Centre Hospitalier Universitaire (CHU) de Québec, where each had been evaluated by a neurologist specializing in neurodegenerative language disorders. Diagnostic classification followed the consensus guidelines proposed by Gorno-Tempini et al.^1^ In accordance with these recommendations, each patient underwent structural or functional neuroimaging—CT (n = 4), MRI (n = 3), or PET (n = 7)—to support diagnostic interpretation, and a lumbar puncture was performed in four cases to further clarify the underlying pathology. Demographic and neuropsychological characteristics of the lvPPA group are presented in Table 1.

**Table 1.** Demographic characteristics and neuropsychological performance of participants in the lvPPA and control groups.

	HC (n = 28)		lvPPA (n = 14)		*F*	*p*	*ηp^2^*
	M (SD)	Min-Max	M (SD)	Min-Max			
*Demographics*							
Age	69.79 (7.70)	53–82	69.93 (7.59)	55–82	0.003	0.955	0.00008
Sex (male/female)	12/16	-	6/8	-		1.00	-
Education (years)	14.68 (2.21)	10–19	14.93 (2.43)	11–18	0.112	0.740	0.0028
Time from diagnosis (months)	-	-	8.43 (6.27)	2–15			
*General cognitive screening*							
MoCA	27.04 (1.90)	24–30	13.79 (6.42)	5–26	103.49	<0.001	0.721
DTLA	93.93 (2.72)	90–98	69.93 (17.08)	40–96	53.86	<0.001	0.574
*Verbal short-term and working memory*							
Short-term memory (digit span forward)	5.93 (0.72)	4–7	4.29 (1.20)	3–6	30.80	<0.001	0.435
Working memory (digit span backward)	4.36 (0.68)	3–6	2.36 (1.08)	0–4	54.02	<0.001	0.575
*Executive functions*							
Alphaflex Time A (sec.)	7.86 (1.51)	6–11	21.85 (27.47)	7–110	7.43	0.010	0.157
Alphaflex Error A	0.39 (0.74)	0–3	4.23 (6.29)	0–18	10.43	0.003	0.207
Alphaflex Time B (sec.)	21.71 (3.78)	15–30	34.62 (22.70)	20–90	8.77	0.005	0.180
Alphaflex Error B	0.86 (.85)	0–3	4.77 (4.71)	0–12	18.55	<0.001	0.317

Values represent means (standard deviations) and ranges. Group differences were assessed using one-way ANOVAs for continuous variables and χ^2^ tests for categorical variables. Effect sizes are reported as partial eta squared (ηp^2^). Higher scores indicate better performance except for Alphaflex completion times (Time A and Time B) and error counts, for which lower values reflect better performance. For interpretation of ηp^2^, values of approximately 0.01, 0.06, and 0.14 are typically considered small, medium, and large effects, respectively.

A comprehensive medical and psychiatric history was obtained for every participant. Individuals were excluded if they reported neurological or cerebrovascular conditions other than lvPPA, any current or past psychiatric disorder as defined by DSM-5,^20^ traumatic brain injury, untreated medical or metabolic conditions (e.g., diabetes, thyroid dysfunction), prior intracranial surgery, or uncorrected auditory or visual impairments.

The comparison group included 28 neurologically healthy adults (twelve men, sixteen women) with a mean age of 69.79 years (SD = 7.70) and an average of 14.68 years of education (SD = 2.21). These participants were recruited through the Centre de recherche CERVO. Exclusion criteria for the control group were: (1) a Z score below −1.33 on the Montreal Cognitive Assessment (MoCA),^21^ based on normative standards established by the research team^22^; (2) a score below the cutoff on the Detection Test for Language Impairments in Adults and the Aged (DTLA) set at the fifth percentile (≈ 1.5 SD below the mean)^23,24^; (3) unstable active psychiatric disorder; (4) history of moderate or severe traumatic brain injury; (5) history of stroke or neurological disease; (6) history of psychotic illness; (7) history of substance abuse; and (8) uncorrected hearing or vision deficits. These criteria were implemented to minimize the likelihood of including participants with subtle cognitive impairments that could bias the normative comparisons. As shown in Table 1, the healthy control and PPA groups did not differ significantly in age, sex, or years of education.

All individuals gave written informed consent after receiving a full explanation of the study procedures. Ethical approval was obtained from the Ethics Committee for Sector Research in Neurosciences and Mental Health (Project MP-13-2017-164).

### Materials

#### Cognitive screening measures

Global cognitive functioning was evaluated using the MoCA, a widely used screening tool designed to detect mild cognitive impairment.^21^ The MoCA assesses multiple domains and was administered according to standardized procedures, with a maximum score of 30.

Language abilities were assessed with the DTLA, a brief screening tool specifically developed to detect language impairments associated with neurodegenerative diseases.^24,25^ The DTLA consists of nine concise tasks targeting the domains most frequently affected in these conditions, including picture naming, word and nonword repetition, verbal fluency, spelling and reading, sentence comprehension, semantic processing, and an alphabetization span measuring verbal working memory. These tasks collectively provide a rapid yet sensitive evaluation of receptive and expressive language processes across lexical, phonological, semantic, and syntactic levels.

#### Background cognitive measures

Short-term and working memory abilities were assessed using the Digit Span subtest from the Wechsler Adult Intelligence Scale.^26^ Forward and backward conditions were administered according to standardized procedures. Forward span provided an estimate of simple verbal short-term memory, whereas backward span required both maintenance and manipulation of verbal information and was considered a measure of working memory Scores for each condition reflected the longest correctly reproduced sequence. These measures were included because phonological short-term memory and verbal working memory are core cognitive mechanisms affected in lvPPA^8,27^ and are known to influence several components of numerical processing, particularly mental calculation and transcoding.^28,29^

Executive functioning was further evaluated with the Alphaflex, a brief test of reactive mental flexibility designed to be rapid, easy to administer, and minimally dependent on visuomotor abilities.^30^ The measure consists of two orally administered parts. In Part A, participants recited the alphabet aloud as quickly and clearly as possible, providing a baseline measure of an overlearned verbal routine. Part B required recitation of the alphabet while producing only every second letter (e.g., A…C…E…), thereby inducing an alternation between overt production and subvocalization. This condition places demands on flexibility, inhibitory control, and the ability to maintain an internal sequence. For each part, the examiner recorded the completion time and total errors (omissions and perseverations). The Alphaflex was selected because executive functions—particularly inhibition, sequencing, and cognitive flexibility—play a central role in complex numerical tasks, including multi-step arithmetic, problem solving, and rule-based transcoding.^31^ Given that lvPPA often involves disruptions to phonological-executive control,^32^ assessing these functions was essential for interpreting performance on the dCALQ.

#### dCALQ: screening test for acquired deficits in number processing

Number-processing abilities were assessed using the dCALQ, a standardized French-language screening tool developed to identify acquired impairments in number processing and calculation.^19^ The dCALQ includes subtests that assess the primary processing components involved in recognizing, producing, and manipulating numerical information. The battery comprises 20 subtests, grouped into three major domains: (1) Recognition and comprehension of digits and numbers; (2) Digit and number production abilities and (3) Calculation processes. A detailed overview of the structure and content of the dCALQ is presented in Table 2.

**Table 2.** Overview of the dCALQ subtests, domains, and scoring.

Domains and subtests	Description	Scoring
**1. Recognition and understanding of digits and numbers**		
Recognition of Arabic digits	Identify visually presented Arabic numerals	10
Recognition of written verbal digits	Identify visually presented number words	10
Recognition of spoken digits	Identify auditorily presented number words	10
Grammaticality judgement on spoken numbers	Decide whether number sequences follow syntactic rules	10
Magnitude comparison of Arabic digits and numbers	Select the larger of two Arabic numbers	10
Magnitude comparison of non-symbolic numbers	Select the larger of two pairs of point clouds	8
Semantic questionnaire on numerical knowledge	True/false questionnaire including general approximate and encyclopedic knowledge	6
*Total score for recognition and comprehension abilities*		*64*
**2. Production of digits and numbers**		
Reading of Arabic numerals	Read aloud Arabic numbers	10
Writing to dictation of Arabic numerals	Convert spoken number words into Arabic numerals	10
Arabic to written verbal transcoding	Convert Arabic numbers into number-word format	5
Written verbal to Arabic transcoding	Convert number words into Arabic numerals	5
*Total score for production abilities*		*30*
**3. Calculation processes**		
Production of mathematical symbols	Write arithmetic symbols to dictation (+, –, ×, ÷, =)	5
Mental addition	Solve orally presented addition problems	5
Mental subtraction	Solve orally presented subtraction problems	5
Mental division	Retrieve/produce multiplication facts	5
Mental multiplication	Perform division problems mentally	5
Word-problem solving	Solve verbally presented problem-solving items	4
*Total score for calculation processes*		*29*
**dCALQ total score**		**123**

The dCALQ yields individual subtest scores, three domain scores, and a total score (maximum = 123). Scoring follows a simple one-point-per-item format, and administration typically requires 20 to 30 min, consistent with the standardized protocol. Higher scores indicate better performance, whereas errors in one or more subtests provide clinically relevant information about specific breakdowns in number processing. The battery is particularly well suited for detecting acalculia in individuals with neurological disorders and has demonstrated strong content and face validity through expert review and pilot testing.^19^

### Procedure

Participants were tested individually in either their home environment or a designated assessment room at the research center. All evaluations were administered by trained research personnel under the oversight of the principal investigator. Each participant completed the full assessment battery in a single session of approximately 45 to 60 min.

The order of task administration was identical for all participants and was selected to limit carryover effects between tasks. Cognitive and language screening measures (MoCA and DTLA) were administered first, followed by the digit span tasks and the Alphaflex to assess verbal working memory and executive control. The session then proceeded with the written word production battery.

Performance was scored during testing using standardized criteria, and all written responses were transcribed verbatim for later qualitative classification of error types. Test data were subsequently entered into a secure database for analysis. Recruitment and data collection were conducted between June 2023 and September 2025.

### Statistical analyses

All statistical analyses were conducted using Jamovi (version 2.6.45).^33^ Statistical significance was set at p < 0.05 (two-tailed) for all analyses.

Descriptive statistics were computed to characterize demographic, cognitive, and numerical performance variables. The distribution of dCALQ scores in the lvPPA group deviated from normality; therefore, non-parametric statistical methods were used for primary group comparisons. Differences between the lvPPA and control groups on dCALQ subtests, domain scores, and total scores were examined using Mann–Whitney U tests. Effect sizes were reported as rank-biserial correlations.

To examine within-group variation in numerical domains among participants with lvPPA, Friedman tests were conducted, followed by Wilcoxon signed-rank tests for post hoc comparisons when appropriate. Associations between numerical performance and background cognitive measures (digit span forward and backward, Alphaflex indices) were assessed using Spearman rank-order correlations.

Receiver operating characteristic (ROC) curve analyses were performed to evaluate the diagnostic accuracy of the dCALQ domain scores and total score in distinguishing lvPPA participants from healthy controls. Additional ROC analyses were conducted for commonly used cognitive screening and background measures (MoCA, DTLA, digit span, and Alphaflex indices) to provide comparative benchmarks. Area under the curve (AUC), optimal cutoff scores (Youden's J index), sensitivity, and specificity were calculated for all measures.

To assess whether group differences in numerical cognition could be explained by overall disease severity or language impairment, additional analyses of covariance (ANCOVA) were conducted using dCALQ total score as the dependent variable, group as the fixed factor, and either MoCA or DTLA scores as covariates. Assumptions underlying ANCOVA (linearity, homogeneity of regression slopes, and normality of residuals) were examined and no major violations were observed. These analyses allowed evaluation of whether numerical impairment remained significant after controlling for global cognitive status and language functioning.

## Results

### Group differences on the dCALQ subtests and numerical domains

Performance on the dCALQ subtests, domain scores, and total score for the lvPPA and control groups is presented in Table 3. Given the non-normal distribution of scores in the lvPPA group, non-parametric Mann–Whitney U tests were used for group comparisons.

**Table 3.** Performance of lvPPA and control groups on the dCALQ subtests, numerical domains, and total score.

Tasks and numerical domains	HC mean (SD)	LvPPA—mean (SD)	*U*	*p*	RBC (HC > lvPPA)
**1. Recognition and comprehension of digits and numbers (64)**	62.96 (1.07)	60.36 (2.87)	322	0.001***	0.643
Recognition of Arabic digits (10)	10.00 (0.00)	9.86 (0.54)	210	0.173	0.071
Recognition of written verbal digits (10)	10.00 (0.00)	9.93 (0.27)	210	0.173	0.071
Recognition of spoken digits (10)	9.93 (0.26)	9.86 (0.36)	210	0.479	0.071
Grammaticality judgement on spoken numbers (10)	9.75 (0.52)	8.36 (1.95)	302.5	0.001***	0.543
Magnitude comparison of Arabic digits and numbers (10)	9.96 (0.19)	9.93 (0.27)	203	0.638	0.036
Magnitude comparison of numbers in analog code (8)	7.36 (0.78)	6.86 (0.77)	264	0.054	0.347
Semantic questionnaire on numerical knowledge (6)	5.96 (0.19)	5.57 (0.51)	273	0.002**	.393
**2. Digit and number production abilities (30)**	29.82 (0.48)	25.43 (6.45)	335	<0.001***	0.709
Reading of Arabic numerals (10)	10.00 (0.00)	8.57 (2.44)	294	<0.001***	0.500
Writing to dictation of Arabic numerals (10)	10.00 (0.00)	8.93 (2.79)	238	0.013*	0.214
Arabic to written verbal transcoding (5)	4.96 (0.19)	3.29 (1.73)	332	<0.001***	0.694
Written verbal to Arabic transcoding (5)	4.86 (0.36)	4.64 (0.63)	226	0.248	0.153
**3. Calculation processes (29)**	28.89 (0.32)	21.07 (7.25)	358	<0.001***	0.827
Production of mathematical symbols (5)	5.00 (0.00)	3.64 (2.24)	252	0.004**	0.286
Mental addition (5)	4.96 (0.19)	4.50 (0.94)	246	0.019*	0.255
Mental subtraction (5)	5.00 (0.00)	4.00 (1.30)	294	<0.001***	0.500
Mental division (5)	5.00 (0.00)	3.07 (1.82)	336	<0.001***	0.714
Mental multiplication (5)	4.96 (0.19)	3.36 (1.60)	318.5	<0.001***	0.625
Word problem solving (4)	3.96 (0.19)	2.50 (1.23)	333	<0.001***	0.699
**dCALQ Total score (123)**	121.68 (1.25)	94.86 (20.70)	388.5	<0.001***	0.982

Values represent means (standard deviations). Mann–Whitney U tests were used due to non-normal distributions in the lvPPA group. Effect sizes correspond to rank-biserial correlations (RBC), with positive values indicating better performance in the control group. Significance levels are indicated as follows: *p < 0.05, **p < 0.01, **p < 0.001.

#### Recognition and comprehension of digits and numbers

The lvPPA group showed reduced performance relative to controls on the overall recognition and comprehension domain score. Within this domain, significant group differences emerged for the grammaticality judgment of spoken numbers and the semantic questionnaire assessing numerical knowledge. Group performance did not differ significantly for recognition of Arabic digits, written number words, spoken number words, or magnitude comparison tasks.

#### Digit and number production abilities (transcoding)

Group differences were also observed in the domain assessing number production abilities, with the lvPPA group scoring significantly lower than controls. At the subtest level, significant impairments were noted for reading Arabic numerals, writing Arabic numerals to dictation, and Arabic-to-written verbal transcoding. No reliable group difference was found for written verbal–to–Arabic transcoding.

#### Calculation processes

The largest group difference was observed for the calculation domain, with the lvPPA group performing markedly worse than controls. Significant deficits were found across all calculation subtests, including mathematical symbol production, mental addition, mental subtraction, mental division, mental multiplication, and word-problem solving.

#### Total score

Consistent with these findings, the lvPPA group obtained significantly lower scores on the dCALQ total score compared to controls, reflecting substantial global impairment in number processing and calculation.

### Intra-group domain profile in lvPPA

To examine whether the severity of numerical impairment varied across components of the dCALQ, a Friedman test was conducted on the three domain scores: (1) recognition and comprehension, (2) transcoding, and (3) calculation. The analysis revealed a significant main effect of domain, *χ^2^*(2) = 25.78, *p* < 0.001, indicating that performance differed across the three numerical components. Post hoc Wilcoxon signed-rank tests showed that recognition and comprehension scores were significantly higher than both transcoding (*W* = 0, *p* < 0.001, *r* = 0.88) and calculation (*W* = 0, *p* < 0.001, *r* = 0.88). Transcoding scores were also significantly higher than calculation scores (*W* = 8, *p* = 0.009, *r* = 0.75). These findings indicate marked differences in performance across the three domains within the lvPPA group.

#### Associations between background measures and numerical performance

Correlation analyses conducted within the lvPPA group revealed three significant associations. Backward digit span was positively correlated with performance in the transcoding (*ρ* = 0.61, *p* = 0.021) and calculation domains (*ρ* = 0.64, *p* = 0.015). In addition, the number of errors produced in the Alphaflex condition B was negatively associated with transcoding scores (*ρ* = –0.65, *p* = 0.015). No other correlations between background measures and dCALQ domains reached significance.

To examine whether group differences in numerical cognition were explained by overall disease severity or language impairment, additional analyses of covariance were conducted controlling for MoCA and DTLA scores. The group effect on dCALQ total performance remained significant after controlling for both MoCA (*p* = 0.021) and DTLA (*p* = 0.027), indicating that numerical impairment in lvPPA cannot be fully explained by global cognitive decline or language deficits alone.

### Diagnostic accuracy of the dCALQ: ROC analyses

**Figure 1.**
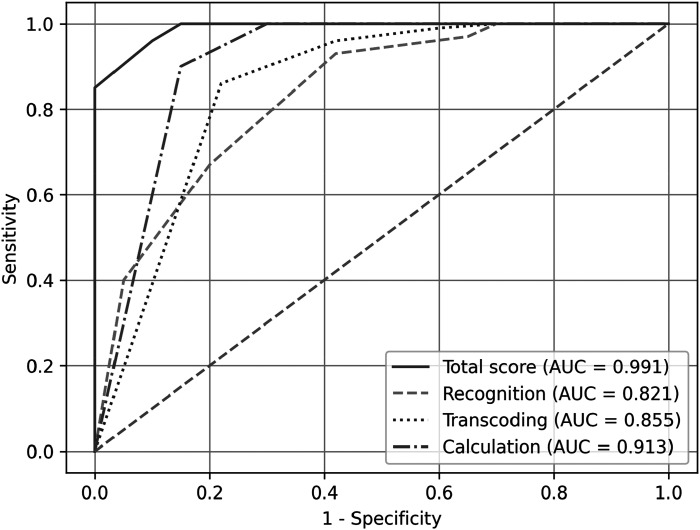
ROC curves for dCALQ total score and domains.

ROC analyses were conducted to evaluate the diagnostic performance of the dCALQ total score and its three domains in distinguishing lvPPA from healthy adults. As shown in Figure 1, the ROC curves indicated excellent discrimination for the total score and strong classification accuracy for both the calculation and transcoding domains, with the recognition/comprehension domain showing more moderate discriminatory ability. Table 4 reports the corresponding AUC values, optimal cutoffs, and classification indices, along with the proportion of lvPPA participants falling below each cutoff.

**Table 4.** ROC indices and proportion of impaired lvPPA participants for the dCALQ total score and domains.

Domain	AUC	Optimal threshold	Sensitivity	Specificity	% lvPPA impaired
Total score	0.991	120.00	0.964	0.929	92.9
Recognition	0.821	62.00	0.929	0.571	57.1
Transcoding	0.855	30.00	0.857	0.786	78.6
Calculation	0.913	29.00	0.893	0.857	85.7

AUC: area under the ROC curve. Optimal cutoffs were determined using Youden's J index. Sensitivity and specificity represent true-positive and true-negative classification rates. Percent impaired indicates the proportion of lvPPA participants scoring below each cutoff.

Additional ROC analyses were conducted for commonly used screening and background measures (MoCA, DTLA, digit span, and Alphaflex). The dCALQ total score showed the highest discriminative performance (AUC = 0.991), exceeding backward digit span (AUC = 0.941). MoCA (AUC = 0.966) and DTLA (AUC = 0.948) also demonstrated excellent discrimination, whereas Alphaflex indices showed only modest classification accuracy (AUCs ≈ 0.74–0.75).

## Discussion

The present study provides the first systematic characterization of numerical cognition in the lvPPA using a comprehensive and theory-driven screening tool. Through detailed analyses of the dCALQ, we identified a robust and graded pattern of impairment across numerical domains, with calculation abilities being most severely affected, followed by number production, and—more mildly—recognition and comprehension of numbers. These deficits were consistently observed across a majority of subtests and were accompanied by high levels of diagnostic accuracy, demonstrating that numerical impairments are a reliable and clinically meaningful feature of lvPPA. Together, these findings deepen our understanding of the broader cognitive profile of lvPPA and are consistent with the involvement of parietal-based numerical processes frequently reported in this syndrome.

One of the central findings is the extent to which numerical performance is degraded in lvPPA relative to healthy controls. Although impaired recognition and comprehension abilities were observed in more than half of the patients, deficits in number production and, especially, calculation were far more pronounced. This pattern of milder deficits in basic recognition, more substantial difficulties in transcoding, and profound impairments in calculation, mirrors the hierarchical organization of numerical cognition, in which increasingly complex processes depend not only on semantic representations of quantity but also on phonological, mnemonic, and executive resources.^10,17,34,35^ Reduced performance in calculation tasks, including mental arithmetic and word-problem solving, suggests that lvPPA affects not only linguistic access to number words but also manipulation of numerical information.

The intra-group profile further clarifies this hierarchical impairment. Even within the lvPPA group, calculation scores were markedly lower than transcoding scores, which were themselves lower than recognition and comprehension scores. This graded pattern suggests that degeneration may affect numerical cognition along a continuum of complexity, with the most integrative and attentionally demanding components being the most vulnerable. This interpretation is consistent with neuroimaging studies reporting prominent involvement of the angular and supramarginal gyri in lvPPA,^3,8^ regions that have been shown to support arithmetic fact retrieval, procedural calculation, and symbolic manipulation.^36,37^ While behavioral in nature, the present findings align with this broader neurocognitive framework.

Although lvPPA is defined clinically as a language-led neurodegenerative disorder, impairment of phonological short-term memory is a well-established and defining feature of the syndrome, consistent with the characteristic difficulties in phrase and sentence repetition observed in these patients.^27,32,38^ Recent work further suggests that semantic control difficulties may also emerge in lvPPA beyond core phonological impairment,^39^ indicating that performance on certain verbally mediated numerical tasks may reflect interactions between numerical processing and broader language-related cognitive mechanisms. The present correlational analyses reinforce this view. Backward digit span, an index of phonological working-memory manipulation, correlated with both transcoding and calculation abilities, but not with recognition or comprehension. Importantly, additional covariance analyses controlling for global cognitive status (MoCA) and language performance (DTLA) confirmed that group differences in numerical cognition remained significant, suggesting that these numerical impairments cannot be fully explained by overall disease severity or language dysfunction alone. This pattern supports previous findings showing that working memory contributes to numerical operations requiring symbolic transformation and multi-step processing, with stronger effects on tasks that rely on the temporary maintenance and manipulation of information.^40,41^ Accordingly, numerical impairment in lvPPA likely reflects the combined impact of phonological working memory constraints, executive demands, and number-processing vulnerabilities. Importantly, because many of the numerical tasks included in the dCALQ involve verbal input or mental calculation, these impairments are best interpreted as reflecting interactions between numerical and language-related processes rather than strictly non-linguistic deficits, consistent with growing evidence highlighting close links between language and mathematical cognition.^42,43^

Similarly, errors in the more demanding condition of the Alphaflex (Part B), which taxes inhibitory control and the ability to maintain an internal alternating sequence, were strongly associated with transcoding performance. This relationship supports the hypothesis that symbolic number production relies not only on stored numerical knowledge but also on executive control processes that guide the application of rule-based transformations.^44,45^ This suggests that executive dysfunction may contribute to numerical impairment beyond phonological mechanisms.

Taken together, these findings suggest that numerical impairments in lvPPA may be related to the broader cognitive consequences of the neurodegenerative process, including phonological, executive, and possibly parietal-network involvement described in previous neuroimaging studies. Neuroimaging studies have shown that arithmetic fact retrieval and multi-step numerical operations rely on coordinated activity within parietal and prefrontal regions,^9,46^ areas commonly affected in lvPPA; thus, the behavioral profile observed here appears broadly consistent with this neurocognitive framework.

The ROC analyses underscore the strong clinical utility of the dCALQ for detecting numerical impairments in lvPPA. The total score achieved near-perfect discrimination between patients and healthy controls, and both the calculation and transcoding domains exhibited excellent diagnostic accuracy. Importantly, the proportion of patients scoring below the optimal cutoff was high across domains, most notably in calculation and transcoding. Additional ROC analyses comparing the dCALQ with commonly used screening and background measures (MoCA, DTLA, digit span, and executive indices from the Alphaflex) further support this interpretation. Although global cognitive and language screening tools also showed high diagnostic accuracy, the dCALQ total score provided the strongest discrimination, suggesting that structured assessment of numerical cognition may capture complementary, and potentially more syndrome-specific, information beyond general cognitive screening or working-memory measures alone.

These findings indicate that numerical impairment appears to be a consistent feature of lvPPA. Clinically, the dCALQ may serve as an efficient screening tool to detect early numerical impairment, monitor disease progression, or support differential diagnosis between lvPPA and other neurodegenerative conditions. To facilitate interpretation of individual variability and relationships between numerical and cognitive measures, individual participant data on the main dCALQ domains together with relevant cognitive indices are provided in Supplemental Table 1. The dCALQ may also assist in differentiating numerical profiles across PPA variants. In the semantic variant, numerical difficulties typically reflect degradation of conceptual number knowledge, affecting magnitude comparison and semantic processing of numerals.^47^ In contrast, the nonfluent/agrammatic variant is characterized primarily by speech production and grammatical deficits, and the available literature does not describe a systematic profile of calculation or number-processing impairment in this syndrome.^2,48^ The structured profile observed in lvPPA, marked impairment in transcoding and calculation with relatively preserved number recognition, differs from both patterns, suggesting that the dCALQ may help delineate syndrome-specific numerical signatures within the PPA spectrum. Beyond diagnostic implications, delineating domain-specific numerical impairments may help clinicians anticipate functional difficulties and guide individualized supportive strategies when numerical deficits meaningfully affect daily functioning. Future studies including larger cohorts across different PPA variants will be important to confirm whether the dCALQ reliably captures distinct numerical profiles between subtypes and to further establish its potential utility for differential diagnosis.

Our results enrich the current understanding of lvPPA as a temporo-parietal syndrome characterized by deficits that extend beyond language. Prior research has primarily emphasized phonological and lexical impairments,^49,50^ but growing evidence indicates that cognitive functions governed by adjacent parietal and fronto-parietal circuits, including praxis,^51^ visuospatial processing,^52^ and executive control,^53^ may also be affected. It is also important to recognize the phenotypic heterogeneity of lvPPA, particularly regarding the relative prominence of phonological versus lexical-semantic impairment, which may reflect variability in the extent of temporo-parietal involvement. Such variability could plausibly contribute to individual differences in numerical and calculation performance, an issue that warrants further investigation in future studies. The present findings suggest that numerical deficits in lvPPA are systematic, measurable, and tightly tied to the cognitive architecture of the syndrome. While calculation impairments are expected given the involvement of the left inferior parietal cortex, the presence of significant deficits in components such as number production underscores the contribution of phonological and executive breakdowns. The selective preservation of magnitude comparison and basic digit recognition in many patients also provides important insights: early numerical semantic knowledge may remain relatively intact, whereas symbolic transformation and calculation processes—those requiring more elaborate integration—appear disproportionately compromised.

Several limitations should be considered when interpreting these findings. First, the sample size is modest, reflecting the rarity of lvPPA but nonetheless limiting statistical power for some analyses. Replication in larger or multicentric cohorts would strengthen the generalizability of the conclusions. Second, the cross-sectional nature of the study precludes inferences about the progression of numerical impairment; longitudinal data are needed to clarify whether calculation declines parallel language deterioration or follow an independent trajectory tied to parietal degeneration. Third, although the dCALQ provides a detailed and reliable measure of numerical processing, future studies incorporating complementary experimental tasks and neuroimaging measures would help further delineate the cognitive and neural mechanisms underlying the observed deficits. In the absence of direct neuroimaging correlates or comparison groups with distinct atrophy patterns, the neuroanatomical interpretation of these numerical deficits should therefore be considered tentative. Finally, because the dCALQ calculation tasks relied exclusively on mental arithmetic, the contribution of visuospatial processing, often engaged during written calculation, was not examined and warrants further investigation.

### Conclusion

This study provides the first systematic investigation of number processing in individuals with the lvPPA. The findings demonstrate that numerical cognition in lvPPA is profoundly and differentially impaired, with calculation abilities being the most severely affected. These deficits appear closely linked to the interaction between phonological working memory, executive processes, and degeneration affecting left temporo-parietal networks. The dCALQ emerges as a highly sensitive tool for detecting numerical impairments in this population and may contribute to improved clinical characterization and differential diagnosis. More broadly, these results underscore the importance of examining non-linguistic cognitive domains in lvPPA to achieve a more comprehensive understanding of the syndrome's neurocognitive architecture.

## Supplemental Material

sj-xlsx-1-alz-10.1177_13872877261450931 - Supplemental material for Beyond language: A structured profile of number processing impairment in logopenic primary progressive aphasia Supplemental material, sj-xlsx-1-alz-10.1177_13872877261450931 for Beyond language: A structured profile of number processing impairment in logopenic primary progressive aphasia by Joël Macoir, Monica Lavoie and Robert Laforce in Journal of Alzheimer's Disease
